# Analysis of CXCR5^+^Th17 cells in relation to disease activity and TNF inhibitor therapy in Rheumatoid Arthritis

**DOI:** 10.1038/srep39474

**Published:** 2016-12-22

**Authors:** Deepika Singh, Matthew Henkel, Bernadette Sendon, June Feng, Anthony Fabio, Diana Metes, Larry W. Moreland, Mandy J. McGeachy

**Affiliations:** 1 Division of Rheumatology, Department of Medicine, University of Pittsburgh, 3500 Terrace St, Pittsburgh, PA 15261, USA; 2 Epidemiology Data Coordinating Center, Graduate School of Public Health, University of Pittsburgh, 130 DeSoto St, Pittsburgh, PA 15261, USA; 3 Department of Surgery, Starzl Transplantation Institute, University of Pittsburgh, PA, USA.

## Abstract

Th17 and TfH cells are thought to promote tissue inflammation and autoantibody production, respectively, in autoimmune diseases including rheumatoid arthritis (RA). TfH cells that co-express Th17 markers (CXCR5^+^Th17) encompass both of these pathogenic functions, and are increased in some human autoimmune settings including juvenile dermatomyositis. We investigated CXCR5^+^Th17 cells in RA subjects with stable or active disease and before and after TNF inhibitor therapy. CXCR5^+^Th17 cell frequency was increased in RA compared to healthy controls, but other helper T cell subsets were not different. CXCR5^+^Th17 cells correlated with disease activity in subjects with active RA prior to initiation of TNF inhibitor therapy. Baseline CXCR5^+^Th17 cells also correlated with numbers of swollen joints as late as one year post-therapy. CXCR5^+^Th17 cell frequencies were unaltered by TNF blockade and in fact remained remarkably stable within individuals. We conclude that CXCR5^+^Th17 cells are not a direct target of TNF blockade and therefore cannot serve as a biomarker of current disease activity. However, basal CXCR5^+^Th17 cell frequency may indicate underlying differences in disease phenotype between patients and predict ultimate success of TNF inhibitor therapy.

Rheumatoid Arthritis (RA) is a prototypic autoimmune disorder characterized by chronic inflammation and autoantibody production with progressive joint and cartilage destruction^1^. Multiple lines of evidence point to a causative role for T cells and B cells reactive to citrullinated self-proteins from joint tissue, which set up a self-perpetuating inflammatory circuit with activated monocytes and synovial fibroblast-like cells^2^,^3^. Autoantibodies against citrullinated peptides (ACPA) and Fc fragment of IgG or Rheumatoid Factor (RF) are considered diagnostic for classic RA. They are a marker of more aggressive disease, present in 50–80% of diagnosed RA patients, either alone or in combination^1^. However, their levels frequently do not diminish in response to therapy^4^. ACPA production has been shown to precede clinical diagnosis of RA by as much as a decade^5^. Hence, ACPA may serve as an indicator of breakdown of B cell tolerance to citrullinated self-antigens.

Certain HLA alleles such as DRB1*04:01 and DRB1*04:04 are strongly associated with disease susceptibility in RA, implicating T cell activation^6^. More recent genome wide association studies further support a wider role for dysregulation of the adaptive immune system in RA, including co-stimulatory molecules and cytokines^7^. T cells are central drivers of most adaptive responses, since they orchestrate activation of B cells, monocytes, and non-immune tissue-resident cells such as synovial fibroblast-like cells. The CD4^+^ Th17 cell subset has been implicated in the pathogenesis of multiple autoimmune diseases in the last decade, including RA. IL-17, the hallmark Th17 cytokine, is elevated in synovial fluid of arthritic joints, and the number of Th17 cells increases in blood of patients with active RA^8^,^9^,^10^,^11^,^12^,^13^. Aside from IL-17, Th17 cells also produce high levels of other pro-inflammatory cytokines -IFNγ, IL-6, GM-CSF and TNF^14^,^15^. These inflammatory cytokines, particularly TNF, strongly synergize with IL-17 to promote chemokine production, bone erosion and pathogenic tissue remodeling through recruitment and activation of monocytes, synovial fibroblasts and osteoclasts^16^,^17^.

CD4^+^ Follicular helper T (TfH) cells express CXCR5, which promotes their homing into B cell zones in lymphoid tissue where they support B cell activation, proliferation and differentiation into plasma cells and memory B-cells^18^,^19^. Several studies have demonstrated an increase in the frequency of CXCR5^+^TfH cells in peripheral blood in RA^20^,^21^,^22^. Similarly, the predominant TfH effector cytokine, IL-21, has been shown to increase in serum of RA subjects^21^,^23^. Functional aberrations within the TfH population in RA have also been reported^24^. Although peripheral blood CXCR5^+^ T cells have been described as TfH cells and can support antibody production better than CXCR5^−^ cells, these cells lack other markers of “true” TfH cells including PD-1, ICOS. CXCR5^+^ T cells are also present along with B cells in inflamed synovium of RA joints, where high levels of the CXCR5 ligand, CXCL13, are found^25^. Hence, circulating blood CXCR5^+^ cells should not be presumed to only enter lymph nodes.

There are intriguing similarities between TfH and Th17 cells, particularly in humans. Development of both TfH and Th17 cells requires ICOS, the ligand for which is expressed on B cells^26^,^27^,^28^. Both subsets produce IL-21, which acts as an autocrine growth factor in Th17 and TfH development^29^,^30^,^31^,^32^. Cytokines that favor development of human TfH cells *in vitro* also result in co-induction of Th17 cells^33^; in fact, conditions to differentially generate TfH versus Th17 cells have not yet been clearly defined for human T cells. Interestingly, many circulating CXCR5^+^ T cells phenotypically overlap with other T helper subsets, as determined by co-expression of CXCR5 with CCR6 (marker of Th17 cells) or CXCR3 (marker of Th1 cells)^34^. Peripheral blood CXCR5^+^ cells that co-express CCR6 have enhanced capacity for B cell activation compared to CXCR5^+^ cells co-expressing CXCR3, and this corresponds with increased IL-17 and IL-21 production^34^. Furthermore, proportions of CXCR5^+^ cells that express CCR6^+^ (termed CXCR5^+^Th17) are increased in juvenile dermatomyositis and Sjogrens’ syndrome^34^,^35^. CXCR5^+^Th17 cells were also found to be increased in RA subjects compared to healthy controls^36^. In JDM, the ratio of (Th17+Th2)/Th1 cells within the CXCR5^+^ population corresponded to disease activity^34^.

TNF inhibitors (TNFi) are the mainstay of biologic therapy for RA. However, the effect of TNFi therapy on peripheral blood CXCR5^+^Th17 cells has not been investigated in RA. We therefore set out to investigate the relationship of circulating CXCR5^+^Th17 cells to disease activity in RA, as well as to study the effect of TNF inhibitor therapy in a longitudinal study of RA subjects with active disease.

## Results

### CXCR5^+^Th17 cells, but not other T helper subsets, are increased in RA

**Figure 1:**
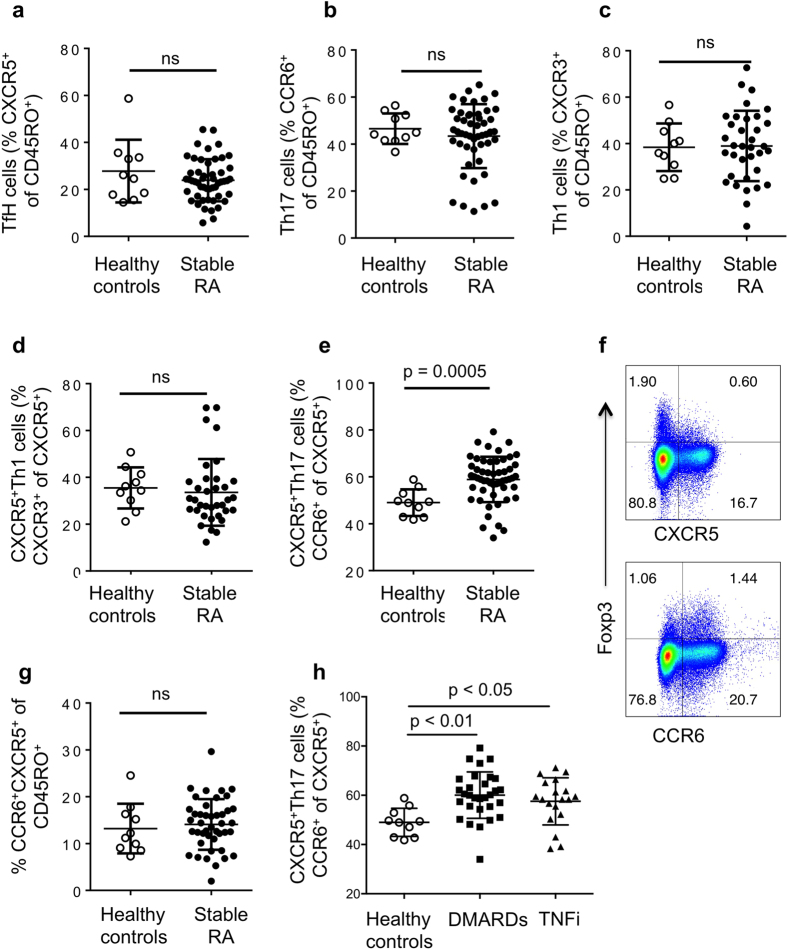
CXCR5^+^Th17 cells, but not other T helper subsets, are increased in RA. PBMC from healthy controls and RA subjects on stable therapy (DMARDs or anti-TNF therapy) for 6 months or longer, as described in Table 1, were analyzed by flow cytometry. (**a**) TfH cells assessed as proportion CXCR5^+^ cells out of CD4^+^CD45RO^+^ cells in; (**b**) Th17 cells assessed as proportion of CCR6^+^ cells out of CD4^+^CD45RO^+^ cells; (**c**) Th1 cells assessed as proportion of CXCR3^+^ cells out of CD4^+^CD45RO^+^ cells; (**d**) CXCR5^+^Th1 cells assessed as proportion of CXCR5^+^ cells that co-express CXCR3; (**e**) CXCR5^+^Th17 cells assessed as proportion of CXCR5^+^ cells that co-express CCR6; (**f**) representative FACS plots for regulatory T cells as assessed by Foxp3 and chemokine receptor expression gated on CD4^+^ T cells in healthy controls (n = 5); (**g**) Proportion of CCR6^+^CXCR5^+^ cells as percentage of CD4^+^CD45RO^+^ cells from healthy controls and RA subjects; (**h**) CXCR5^+^Th17 cells assessed as proportion of CXCR5^+^ cells that co-express CCR6 in healthy controls and RA subjects split according to therapy received. For each graph, each dot represents a unique individual in the study, n for groups indicated in Table 1.

We analyzed proportions of Th1 (CXCR3^+^), Th17 (CCR6^+^) and TfH (CXCR5^+^) cells within the CD4^+^CD45RO^+^ activated/memory population of a cross sectional group of seropositive Rheumatoid Arthritis patients on stable therapy (Cohort 1), compared to age and gender-matched healthy controls. Demographics are shown in Table 1. We also assessed the proportion of TfH cells that co-expressed CXCR3 (CXCR5^+^Th1) or CCR6 (CXCR5^+^Th17) as described by Morita *et al*.^34^. Typical FACS plots and gating strategies are shown in Supplementary Figure 1a. No differences were observed in the proportions of Th1, TfH, Th17 and CXCR5^+^Th1 subsets when comparing RA subjects and healthy controls (Fig. 1a–d). However, CXCR5^+^Th17 cells were significantly increased in RA subjects compared to healthy controls (Fig. 1e).

Chemokine receptors are well-validated markers of T helper subsets, and we confirmed that *ex vivo* frequencies of CCR6^+^ cells and CCR6^+^CXCR5^+^ cells in the PBMC memory population correlated with subsequent IL-17 secretion in a 5 day *in vitro* Th17 stimulation assay cells (Supplementary Figure 1b,c). CXCR5^+^Th17 cells frequencies showed a similar strong trend with IL-17 production that did not quite reach significance most likely due to variations in proportion of total CXCR5^+^ cells (Supplementary Figure 1d). Regulatory T cells are present in the CD45RO^+^ memory population, however the majority of Foxp3^+^ Tregs were consistently CXCR5^–^ while some were CCR6^+^ (Fig. 1f, Supplementary Table 1). Furthermore, the vast majority of CXCR5^+^ and CCR6^+^ cells were in fact Foxp3^−^, indicating that changes in proportions of Tregs are unlikely to contribute to the altered frequencies of CXCR5^+^Th17 cells.

The total CXCR5^+^CCR6^+^ population within memory cells was not different between controls and RA (Fig. 1g), suggesting that the phenotype of the CXCR5^+^ TfH population itself is the important feature as reported previously^34^,^35^. This cohort of RA subjects had stable disease and therapy, and CXCR5^+^Th17 cells were increased in RA subject receiving either non-biologic DMARDs or TNFi (with or without DMARDs as indicated in Table 1) (Fig. 1h).

### T cell subsets do not correlate with disease activity in established RA

**Figure 2:**
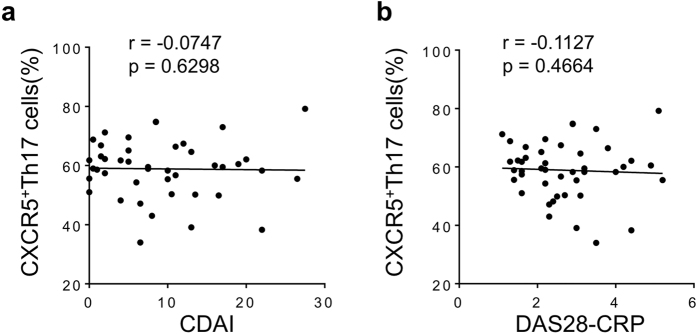
CXCR5^+^Th17 cells do not correlate with disease activity in stable RA. (**a**) Correlation between CXCR5^+^Th17 cells (frequency of CXCR5^+^ cells that express CCR6) and CDAI in stable therapy RA; (**b**) Correlation between CXCR5^+^Th17 cell subset and DAS28-CRP in stable therapy RA. For each graph, each dot represents a unique individual in the study.

We next analyzed if there was a relationship between disease activity and T cell subsets in this cohort. None of the T cell subsets correlated with either clinical disease activity index (CDAI) or Disease Activity Score in 28 joints (DAS28-CRP) (Fig. 2a, b-data shown only for CXCR5^+^Th17 cell subset but similar results observed for Th1, TfH, Th17 and CXCR5^+^Th1 cell subsets).

### CXCR5^+^Th17 cells correlate with baseline disease activity in active RA prior to therapy change

**Figure 3:**
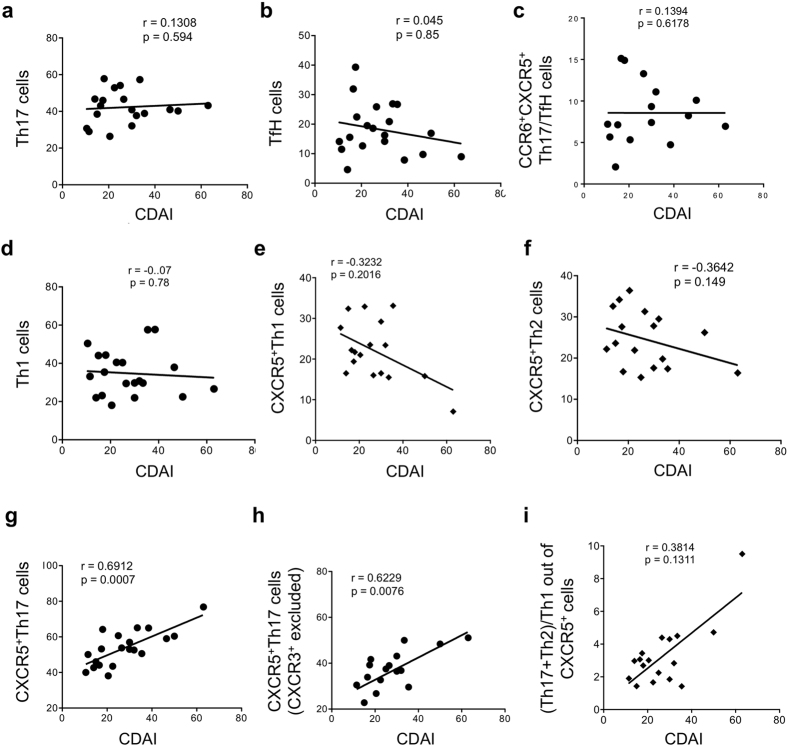
CXCR5^+^Th17 cells correlate with baseline disease activity in active RA prior to therapy change. Correlations between clinical disease activity index (CDAI) and (**a**) Th17 cells as defined in Fig. 1, (**b**) TfH cells, (**c**) CCR6^+^CXCR5^+^ cells, (**d**) Th1 cells, (**e**) CXCR5^+^Th1 cells, (**f**) Th2 cells, (**g**) CXCR5^+^Th17 cells; (**h**) CXCR5^+^Th17 cells excluding CXCR3 expressing cells, (**i**) Ratio of CXCR5^+^Th17 plus CXCR5^+^Th2 cells divided by CXCR5^+^Th1 cells to clinical disease activity. All PBMC subsets and disease activity assessed at baseline prior to initiation of TNF inhibitor, cohort demographics described in Table 1. For each graph, each dot represents a unique individual in the study.

The subjects analyzed so far had well-controlled disease (low CDAI scores) and stable therapy for 6 months or more prior to analysis, supporting stable disease activity, and this may affect the identification of disease-associated patterns. We therefore identified a second cohort of RA subjects with active disease that was uncontrolled by DMARD therapy alone (cohort 2, Table 1), and analyzed blood T cells prior to initiation of anti-TNF therapy. There were no correlations between standard measures of RA disease activity and proportions of Th17 (CCR6^+^) cells, TfH (CXCR5^+^) cells or CCR6^+^CXCR5^+^ memory cells at baseline visit prior to therapy initiation (Fig. 3a–c). There were also no correlations between disease activity and Th1 cells, CXCR5^+^Th1 cells or CXCR5^+^Th2 cells (Fig. 3d–f). However, baseline CXCR5^+^Th17 cell frequencies showed a significant positive correlation with disease activity (Fig. 3g). Excluding CXCR3^+^ cells from the CXCR5^+^Th17 population did not change the results (Fig. 3h). In JDM, the ratios of Th17 and Th2 cells to Th1 within the CXCR5^+^ population was found to correlate with disease activity measures. We applied this analysis approach, and although the trend was similar these ratios did not give a significant correlation with disease activity in our active RA cohort (Fig. 3i). In all cases, similar correlations (and significance) were found with DAS28(crp) as a measure of disease activity (data not shown). Bivariate analyses ruled out gender, age, sex and medications as confounding factors in the relationship between disease activity indices and CXCR5^+^Th17 cell frequencies (data not shown).

### CXCR5^+^Th17 cells do not correlate with RF or CCP autoantibody in serum

**Figure 4:**
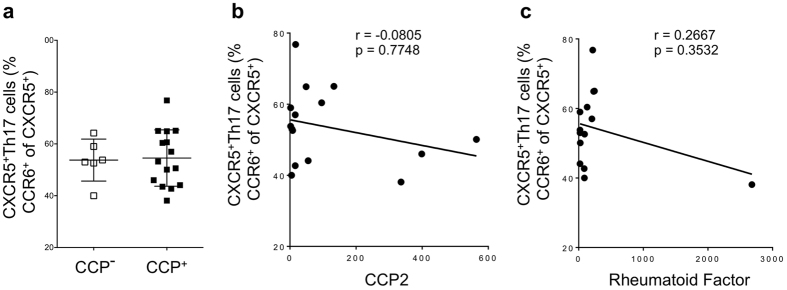
Seropositivity does not influence CXCR5^+^Th17 cell frequency. (**a**) Frequency of CXCR5^+^Th17 cells (gated as CCR6^+^ cells out of CXCR5^+^ CD4^+^CD45RO^+^ cell population) in CCP negative and CCP positive subjects in cohort 2 as described in Table 1. (**b**) Correlation between CCP levels in serum and CXCR5^+^Th17 cells at baseline prior to therapy initiation. (**c**) Correlation between RF levels in serum and CXCR5^+^Th17 cells at baseline prior to therapy initiation. For each graph, each dot represents a unique individual in the study.

It has previously been demonstrated that CXCR5^+^Th17 cells efficiently activate B cells to produce antibody^34^,^36^. We therefore queried whether CXCR5^+^Th17 cell frequencies were associated with seropositivity for RA-associated autoantibody. There was no difference in mean frequencies of CXCR5^+^Th17 cells between CCP+ and CCP- RA subjects (Fig. 4a), and similar results were found for the other T helper subsets analyzed (data not shown). Likewise, CXCR5^+^Th17 cells did not correlate with serum levels of CCP or RF (Fig. 4b,c). We would like to highlight the caveat that these assays were developed for diagnostic purposes and may therefore miss subtleties in autoantibody composition, isotype or specificities not recognized in these assays.

### Subjects with high CXCR5^+^Th17 cells have greater numbers of swollen joints before and after TNF inhibitor therapy

**Figure 5:**
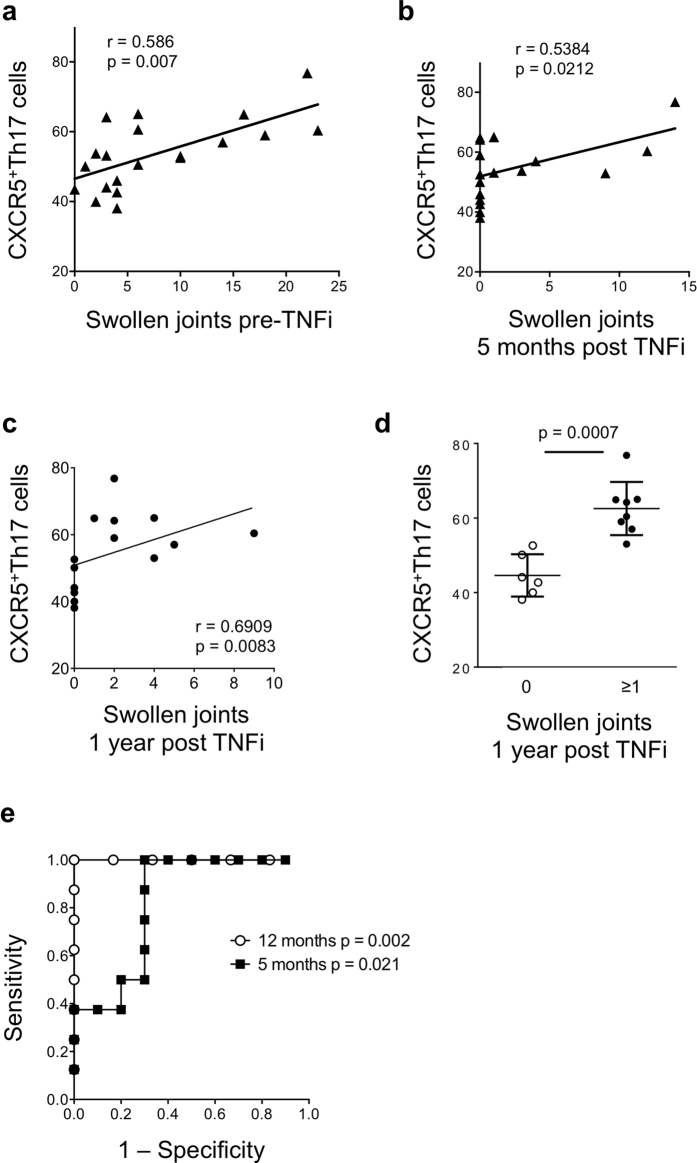
Baseline CXCR5^+^Th17 cells predict swollen joints before and after TNF blockade. (**a**) Baseline CXCR5^+^Th17 cells (as defined in Fig. 1) prior to initiation of TNF inhibitor therapy were correlated with number of swollen joints at baseline; (**b**) Baseline CXCR5^+^Th17 cells were correlated with numbers of swollen joints at 5 months post TNF inhibitor therapy; (**c**) Baseline CXCR5^+^Th17 cells were correlated with numbers of swollen joints at 1 year post TNF inhibitor therapy; (**d**) CXCR5^+^Th17 cell frequency at baseline in subjects with presence or absence of swollen joints at 1 year, bars represent mean ± SD; (**e**) ROC analysis showing sensitivity/specificity of baseline CXCR5^+^Th17 cell frequency in subjects with and without swollen joints at 5 months and 1 year post-TNFi therapy.

We then set out to determine which factors within the composite disease activity scores were most strongly related to the observed immune cell perturbances in RA subjects. High proportions of CXCR5^+^Th17 cells could suggest an increased inflammatory state in individuals with active disease. However, there was no correlation between the systemic inflammatory biomarker C-reactive protein and T cell subsets pre-TNFi therapy (for CXCR5^+^Th17 cells and CRP, Spearman r = 0.1588) or post-TNFi therapy (Spearman r = 0.2208). Similarly to CRP, CXCR5^+^Th17 cells did not correlate with physician or patient global health scores (not shown). T helper cell subset frequencies were also not related to disease duration in this cohort (for example, CXCR5^+^Th17 cells Spearman r = 0.1029 p = 0.7044). However, baseline CXCR5^+^Th17 cell frequencies significantly correlated with the numbers of swollen joints (which is associated with progressive joint damage) prior to TNF inhibitor therapy (Fig. 5a). There was a similar trend with numbers of baseline tender joints although not reaching significance (data not shown).

We next assessed the predictive value of baseline CXCR5^+^Th17 cells for therapy response. CXCR5^+^Th17 cell frequency did not correlate with improvement in disease activity as measured by % change in CDAI (r = −0.265, p = 0.305). This supports the previous finding that CXCR5^+^Th17 cells do not correlate with all measures of this composite disease activity index. However, there was a positive correlation between baseline CXCR5^+^Th17 and swollen joints at 5 months after TNF inhibitor therapy (Fig. 5b), even though absolute numbers of swollen joints decreased for most subjects. Baseline CXCR5^+^Th17 cells again did not correlate with numbers of tender joints at 5 months (r = 0.02709, p = 0.9207). Strikingly, baseline CXCR5^+^Th17 frequencies also correlated with numbers of swollen joints following one year of stable TNF inhibitor therapy (Fig. 5c). Furthermore, subjects without any swollen joints after one year of therapy had a significantly lower proportion of CXCR5^+^Th17 cells prior to therapy initiation (Fig. 5d).

This is admittedly a small cohort, and results need to be validated in a larger group of RA subjects. We performed a preliminary ROC analysis to further analyze the association between CXCR5^+^Th17 cells and presence of swollen joints following therapy (Fig. 5e). At one year post-therapy, ROC analysis indicated a cutoff value for CXCR5^+^Th17 cells of 51.35% gave a likelihood ratio of 6.0, with sensitivity of 1 (95% CI 0.6306-1) and specificity of 0.8333 (95% CI 0.3588–0.9958) for presence of swollen joints. At 5 months, the indicated cutoff was 52.8% with slightly lower likelihood ratio of 3.33, sensitivity of 1 (0.6306–1) and specificity of 0.7 (0.3475–0.9333). These data set predictive values for CXCR5^+^Th17 cell frequencies that can now be validated in independent cohorts of RA subjects with active disease prior to therapy initiation.

### T cell subsets are not altered by TNF inhibitor therapy, and remain stable within individuals

**Figure 6:**
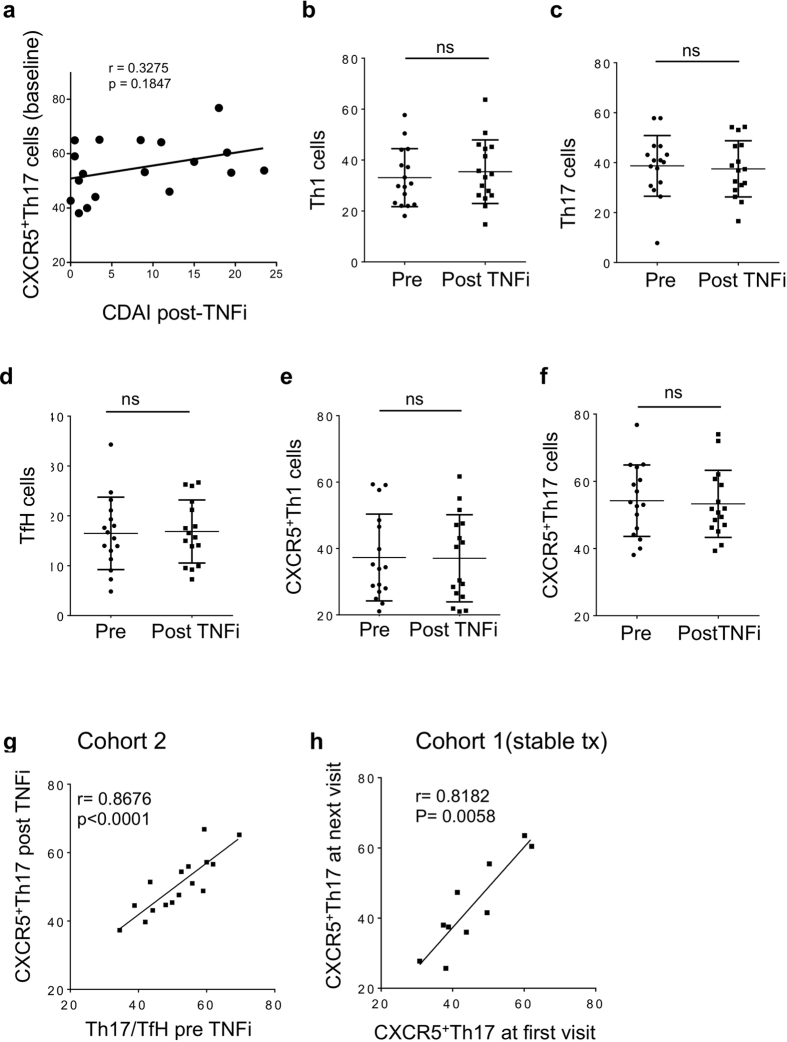
Helper T cell subsets are unaltered by TNF blockade and remain stable within individuals. (**a**,**b**) Correlations between CXCR5^+^Th17 cell subset and CDAI assessed 5 months after anti-TNF therapy; (**b–f**) RA subject blood T cell subsets (as defined in Fig. 1) were analyzed by flow cytometry pre and post therapy with TNF inhibitors; (**g**) Correlation of CXCR5^+^Th17 cell frequency before and after therapy in each subject; (**h**) CXCR5^+^Th17 cells in individual RA subjects from cohort 1 on stable therapy, assessed longitudinally (range 2–23 months). Each data point represents a single subject; bars represent mean ± SD.

The above data demonstrating a positive correlation between CXCR5^+^Th17 and disease activity led us to test whether CXCR5^+^Th17 cells are a direct target of TNFi therapy. There were no correlations between CXCR5^+^Th17 subset and disease activity after TNFi therapy (range 3–8 months) (Fig. 6a), despite significant decrease in disease activity (Table 1). Corresponding to this observation, none of the T cell subsets showed significant changes from baseline following TNF blockade (Fig. 6b–f). Furthermore, it was intriguing that while proportions of T helper cell subsets showed considerable variation *between* individuals (Fig. 5f), there was a remarkable concordance of CXCR5^+^Th17 cell frequencies before and after TNFi therapy *within* individuals (Fig. 5g). This confirms that CXCR5^+^Th17 cell frequencies are not changed by TNF inhibitor therapy, and further suggests that this population is very stable within individuals over time. To confirm this finding, we obtained longitudinal samples from Cohort 1 subjects with stable RA and therapy over several months (range 2–23 months, see Table 1) and observed a similar striking correlation for CXCR5^+^Th17 cell frequency within individuals over time (Fig. 6h). Temporal stability was similarly observed for Th17, TfH and Th1 subsets within the CD4^+^CD45RO^+^ population (data not shown). These findings suggest CXCR5^+^Th17 cells are indicative of underlying ‘pre-set’ pathophysiology rather than indicators of current inflammatory state.

## Discussion

The literature contains conflicting reports on the effects of therapy on Th17 cells in RA. Szalay *et al*. analyzed proportions of CCR6^+^ Th17 cells in subjects with established RA before and after 8 weeks of anti-TNF therapy, and found no significant change in the Th17 population^37^, similar to our findings. In contrast, Lina *et al*. reported that 12 weeks of combined therapy with Methotrexate and Etanercept decreased the proportion of IL-17^+^ T cells and serum IL-17 levels in RA subjects, although correlations between IL-17 and disease activity were not assessed^38^. Both of these studies found increased Tregs following TNF blockade, and therefore an altered Th17/Treg ratio. On the other hand, circulating Th17 cells were reported to increase at 6 months post TNF inhibitor therapy in inadequate responders^12^ and in the blood of previously biologic-naive RA subjects showing a good response to Etanercept or Adalimumab therapy^39^. These studies generally measure chemokine receptors or IL-17 production as a proportion of total CD4^+^ T cells, while we first gated on CD45RO^+^ cells to normalize for proportion of activated/memory T cells between individuals. Taking the balance of these studies together with our own data, we conclude that there is no significant effect of TNF inhibitors on Th17 cell frequencies.

TfH cells have been associated with autoimmune disease, but very little data exists on their role in RA, or the effect of TNF inhibitors on these cells. Zhang *et al*. report decreased serum IL-21 without a change in TfH cell frequency following DMARD therapy^40^. CXCR5^+^Th17 cells were first shown to correlate with autoimmune disease activity in patients with juvenile dermatomyositis^34^. CXCR5^+^Th17 cells were also increased in Sjogren’s syndrome^35^. Our data further support CXCR5^+^Th17 cells as pathogenic players in rheumatoid arthritis. Interestingly, we found that the proportion of total CCR6^+^ cells within CXCR5^+^ cells showed the strongest correlations with disease activity: factoring in Th1 and Th2 proportions in fact decreased the association, in contrast to the findings in JDM. It is important to note that correlations between CXCR5^+^Th17 cells and disease activity came to light when we analyzed a cohort of subjects with active RA as defined by a decision to change to biologic therapy, rather than based on an arbitrary disease score cutoff in subjects with stable disease/therapy who could represent a very different state of disease.

The finding that CXCR5^+^Th17 cell frequency at baseline correlates with swollen joints at baseline and at one year post-TNFi therapy could indicate that patients with high CXCR5^+^Th17 frequencies have more severe joint disease, which is therefore more refractory to therapy. However, the finding that CXCR5^+^Th17 cells are not decreased by TNFi therapy, and that this population is higher overall in stable RA subjects compared to healthy controls show that CXCR5^+^Th17 cells are not a useful blood biomarker of overall disease activity in RA. Rather, these data support further investigation of the association between raised CXCR5^+^Th17 cell frequencies and swollen joints, both as predictors of therapy response and to better understand whether this population could indicate differences in RA disease pathogenesis that could be uniquely targeted in a subset of RA patients.

Arroyo-Villa *et al*. recently reported an increase of CXCR5^+^Th17 cells in patients with RA, and confirmed the enhanced capability of CXCR5^+^Th17 cells to activate B cells^36^. Similar to our findings, they did not find any association between disease activity (DAS28) and any TfH cell subset in their cohort of cross-sectional RA subjects receiving non-biologic DMARDs, the majority of whom had low disease activity. Mirota *et al*. previously reported the enhanced capacity of CXCR5^+^Th17 cells to promote B cell antibody production compared to CXCR5^+^Th1 cells^34^. Although a hallmark feature of RA, the role of antibodies targeting citrullinated proteins remains unclear in disease pathogenesis, since biologic therapy including B cell depletion can be clinically effective without eliminating circulating CCP. We did not find any differences in CXCR5^+^Th17 levels between seropositive or seronegative RA subjects, or correlations between serum CCP/RF and CXCR5^+^Th17 cell frequency. It is possible that these diagnostic assays could miss subtle changes in autoantibody that could change their function/targets. Alternatively, T cell CXCR5 expression may be indicative of T: B cell interactions that drive other pro-inflammatory circuits such as cytokine production in the synovium, or of recruitment to inflamed synovium that expresses chemokine ligands of both CXCR5 and CCR6. The precise roles of CXCR5^+^Th17 cells in RA joint pathology, beyond driving antibody production, therefore warrants further investigation.

TNF inhibitors are thought to predominantly act locally in the joint tissue to block the cycle of immune activation. This does not exclude activation of T cells and B cells in that site, for example through induction of cytokines and costimulatory molecules. However, circulating TfH/Th17 and other T cell subsets did not change with therapy-induced changes in disease activity or markers of acute inflammation such as CRP, and in fact remained quite stable within individuals. This suggests that there is not a large egress of specific T cell subsets into circulation as joint inflammation is quelled. Neither does it support a model in which blood subsets represent ‘spillover’ of activated inflammatory cells from inflamed synovium. Rather, our data suggest that RA subjects who have a heightened CXCR5^+^Th17 immune response may also be less likely to achieve complete remission by TNFi therapies compared to RA that is dominated by other immune pathways (such as synovial fibroblast-macrophage circuits). It is also possible that high CXCR5^+^Th17 cell frequencies result in a different form of joint damage, resulting in remaining joint thickening and swelling after TNFi therapy, despite decreased acute inflammatory measures such as pain and serum CRP. Since joint damage is a main contributor to disability it will be important in future studies to address the types of joint inflammation driven by CXCR5^+^Th17 cells compared to other T cell subsets such as Th1 cells. It would also be important to determine when T helper cell subset frequencies become ‘stablized’ in RA, is this an event that occurs in the at-risk phase prior to disease onset, or during early RA? This would point to potential opportunities for early intervention to alter the course of disease progression in RA subjects. These findings, once validated in larger cohorts, will likely lend support to an earlier and more aggressive therapy approach, including use of biologics, for RA patients presenting with a high CXCR5^+^Th17 signature.

Significant therapeutic advances have been made in RA, particularly in the realm of biologics. However, questions still remain regarding optimal treatment strategies. Fitting with a downstream role of TNF in joint inflammation, CXCR5^+^Th17 cell frequencies are not themselves altered by TNF blockade; however high frequencies of CXCR5^+^Th17 cells correspond to poorer resolution of joint swelling. The Th17-produced cytokine IL-17 is known to show strong synergy with TNF and these data therefore support the approach of combined cytokine blockade for therapy of refractory RA joint inflammation. Early clinical trials exploring neutralization of IL-17A with Secukinumab, a fully human anti-IL-17A monoclonal antibody, found a limited benefit in a subset of patients^41^. Bispecific antibodies targeting IL-17 and TNF are currently in development by multiple pharmaceutical companies^42^, and it would be very interesting to determine the link between CXCR5^+^Th17 cells and response to these therapies.

## Methods

### RA Patient Cohort

Samples were obtained from RA patients enrolled in the Rheumatoid Arthritis Comparative Effectiveness Research (RACER) registry at the University of Pittsburgh after obtaining written informed consent. Data collected in RACER includes age, gender, race/ethnicity, disease duration, disease activity assessed by using CDAI (clinical disease activity index) and DAS28-CRP (C reactive protein); current medications, treatment change as well as laboratory tests including RF, CCP2 antibodies are also measured at each RACER visit. All RACER studies received Institutional Review Board approval at University of Pittsburgh and this study was carried out in accordance with the approved guidelines.

Cohort 1 consisted of 44 RACER subjects on stable therapy with either DMARDs or anti-TNF therapy for a period of 6 months or longer. Cohort 2 included 16 RACER subjects with active disease who were analyzed prior initiation of anti-TNF therapy and at a follow-up visit within 6 months (+/− 2.5 months). We also analyzed 10 healthy controls, without autoimmune disease. Subject characteristics are summarized in Table 1.

### Sample Collection, Preparation, and Storage

For PBMC isolation, peripheral blood was collected in a lithium heparin tube, diluted 1:1 with phosphate buffered saline (Fisher, Hampton, New Hampshire, USA), overlaid with Lymphoprep^TM^ reagent (Axis-Shield, Dundee, Scotland), subjected to room temperature centrifugation for 30 minutes at 800 × g, then the lymphocyte layer was collected in another tube and subjected to two rounds of washing in PBS by room temperature centrifugation for 10 minutes at 300 × g. Isolated PBMC were either used freshly or cryopreserved in 90% heat-inactivated fetal bovine serum (Gemini Bio-Products, West Sacramento, CA, USA) 10% DMSO (Fisher, Hampton, New Hampshire, USA) until batch thawed for immunophenotyping. Healthy controls and Cohort 1 of stable therapy RA patients were analyzed from freshly isolated PBMCs while Cohort 2 subjects were analyzed from the cryopreserved PBMCs. From our observation, the process of freeze-thawing led to an overall slight decrease in T-cell populations and hence Cohort 2 was not included in the comparison with healthy controls.

### *In vitro* Th17 stimulation assay

PBMC were cultured in 96 well plates (200,000 cells/well) in duplicate with superantigen Staphylococcal enterotoxin B (SEB; 1 μg/ml)), anti-human CD28 (CD28.2; 2.5 μg/ml) (Bio Legend), cytokines IL-23 (50ng/ml, R&D Systems) and IL1β (50 ng/ml, R&D Systems) for 5 days. Supernatant was collected and analyzed by ELISA (eBioscience San Diego, CA, USA) for IL-17A secretion. RNA extraction was performed using the QIAGEN^®^ RNeasy Kit, RNA was quantified and purity checked using Agilent 2100 Bioanalyzer, RNA were reverse transcribed using the High Capacity cDNA Reverse Transcription Kit (Applied Biosystems) in a total volume of 20 μL. Samples were analyzed by qRT-PCR for RORγt expression.

### Flow Cytometry

Cohort 1 was analyzed from freshly isolated PBMCs and for Cohort 2 analysis, frozen PBMCs were briefly thawed in 37 °C water bath, then washed in RPMI containing 10% FBS. PBMC were allowed to recover overnight in complete RPMI media then stained with the following fluorescently labeled anti-human antibodies to define T cell subsets: anti-CXCR3 (1C6/CXCR3), anti-CXCR5 (RF8B2), anti-CCR6 (11A9), anti-CD4 (RPA-T4), anti-FoxP3 (259D/C7) (BD Biosciences, San Jose, California, USA) and anti-CD45RO (UCHL1)(eBioscience, San Diego, CA, USA). Data were analyzed using FlowJo software (Flowjo, LLC, Ashland, OR, USA).

### Statistical analysis

Comparisons between continuous data were done using Mann-Whitney t-test or by Wilcoxon signed rank test for paired sample analysis, as indicated; data are expressed as mean ± SD. Significance of correlation was measured using Spearman’s test, P values < 0.05 were considered significant.

## Additional Information

**How to cite this article**: Singh, D. *et al*. Analysis of CXCR5^+^Th17 cells in relation to disease activity and TNF inhibitor therapy in Rheumatoid Arthritis. *Sci. Rep.* **6**, 39474; doi: 10.1038/srep39474 (2016).

**Publisher's note:** Springer Nature remains neutral with regard to jurisdictional claims in published maps and institutional affiliations.

## Supplementary Material

Supplementary Information

## Figures and Tables

**Table 1:** Patient and Control Characteristics.

	Healthy Controls	Cohort 1	Cohort 2
**Description**	Currently healthy, No autoimmune disease	Stable RA, stable DMARD/TNFi therapy	Active RA on DMARD monotherapy, Pre and post TNFi
**Number**	10	44	21 (baseline) 17 (with follow-up)
**Age (years)**	51.6 ± 8.44 Range 37–63	57.27 ± 7.55 Range 31–69	54.77 ± 16.31 Range 25–77
**Gender**	70% females	75% females	85.71% females
**Race**	80% Caucasian	84% Caucasian	90.5% Caucasian
**DAS28-CRP**	NA	2.69 ± 1.06	4.75 ± 1.63 (pre-TNFi)2.52 ± 1.17 (post-TNFi)
**CDAI**	NA	9.13 ± 7.01	27.83 ± 16.66 (pre-TNFi)8.25 ± 8.33 (post-TNFi)
**Interval between visits (months)**	NA	NA	4.85 ± 1.49 Range 3–8
**Medications (%)**			
**DMARD alone**		59.09	—
**TNFi alone**		6.80	19.04
**TNFi** + **DMARD**		34.09	80.95
**Concomitant steroids**		44	38
