# Supplementary material for: Analysis of CXCR5+Th17 cells in relation to disease activity and TNF inhibitor therapy in Rheumatoid Arthritis
Source: Sci Rep. 2016 Dec 22;6:39474. doi: 10.1038/srep39474 (PMC5177940; doi:10.1038/srep39474)
Supplement: Supplementary Information [file srep39474-s1.pdf]

Analysis of CXCR5⁺Th17 cells in relation to disease activity and TNF inhibitor therapy in Rheumatoid Arthritis

Deepika Singh[1], Matthew Henkel[1], Bernadette Sendon[1], June Feng[2], Anthony Fabio[2], Diana Metes[3], Larry W Moreland[1], Mandy J McGeachy[1]*

Supplementary Figure 1

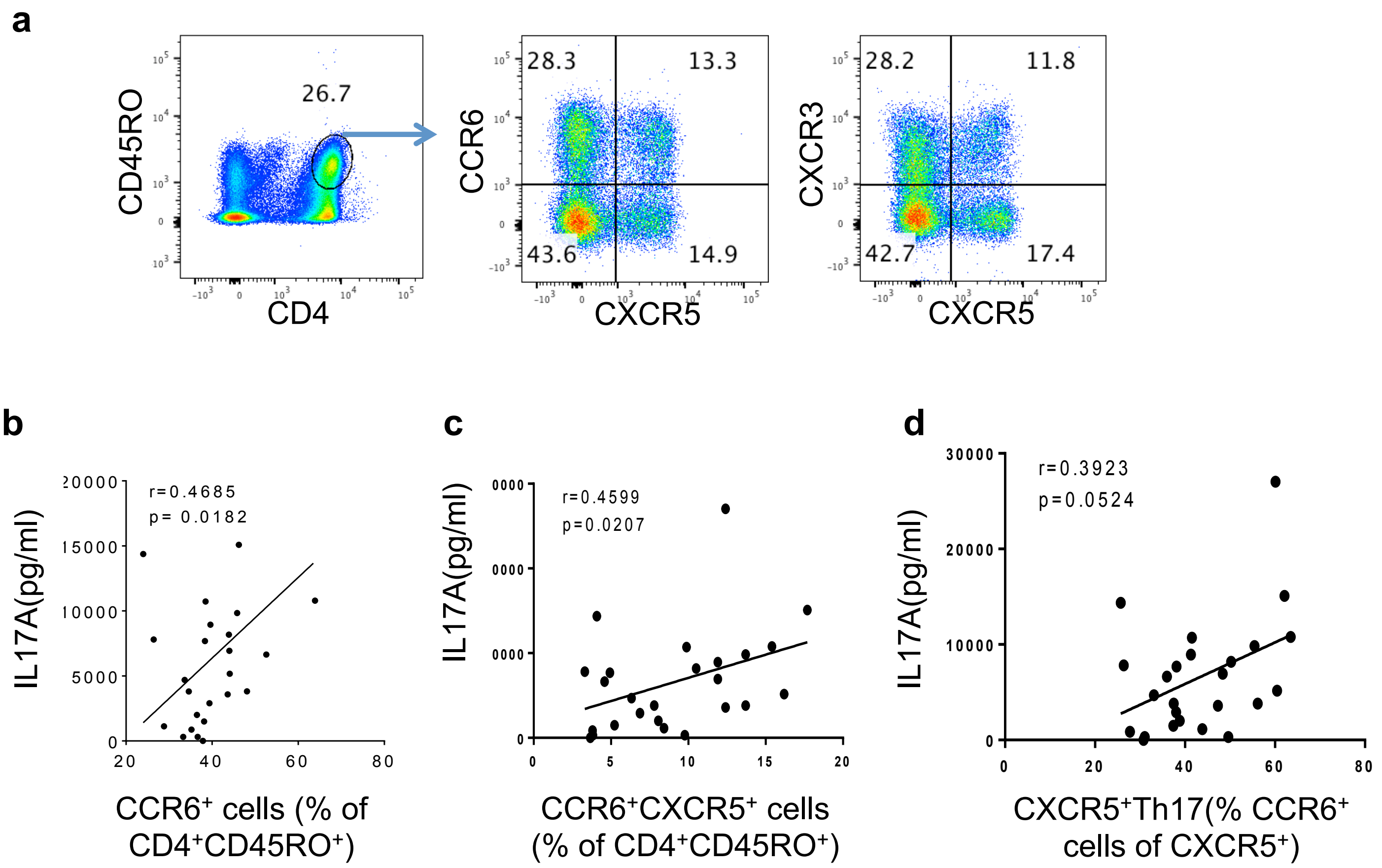

a: Healthy control and RA subject blood T cell subsets were analyzed by flow cytometry, representative FACS staining. **b-d:** PBMC from RA subjects were analyzed ex vivo by FACS as shown in **a**, then cultured for 5 days with SEB, CD28, IL23 and IL1, and IL-17 in supernatants was assessed by ELISA.

Analysis of CXCR5^{+}Th17 cells in relation to disease activity and TNF inhibitor therapy in Rheumatoid Arthritis

Deepika Singh[1], Matthew Henkel[1], Bernadette Sendon[1], June Feng[2], Anthony Fabio[2], Diana Metes[3], Larry W Moreland[1], Mandy J McGeachy[1]*

Supplementary table 1

	Donor 1	Donor 2	Donor 3	Donor 4	Donor 5
FOXP3+of CXCR5+	4%	0.25 %	0.89%	1.63%	0.60%
FOXP3+of CCR6+	4.78%	3.78%	3.27%	3.66%	3.81%
CXCR5+ of FOXP3+	29.22%	4.26%	6.82%	6.93%	6.83%
CCR6+ of FOXP3+	69.15%	76.28%	49.28%	53.61%	63.60%
